# Supplementary material for: Higher Prevalence of Bacteroides fragilis in Crohn’s Disease Exacerbations and Strain-Dependent Increase of Epithelial Resistance
Source: Front Microbiol. 2021 Jun 8;12:598232. doi: 10.3389/fmicb.2021.598232 (PMC8219053; doi:10.3389/fmicb.2021.598232)
Supplement: Supplementary file 1 [file Image_1.pdf]

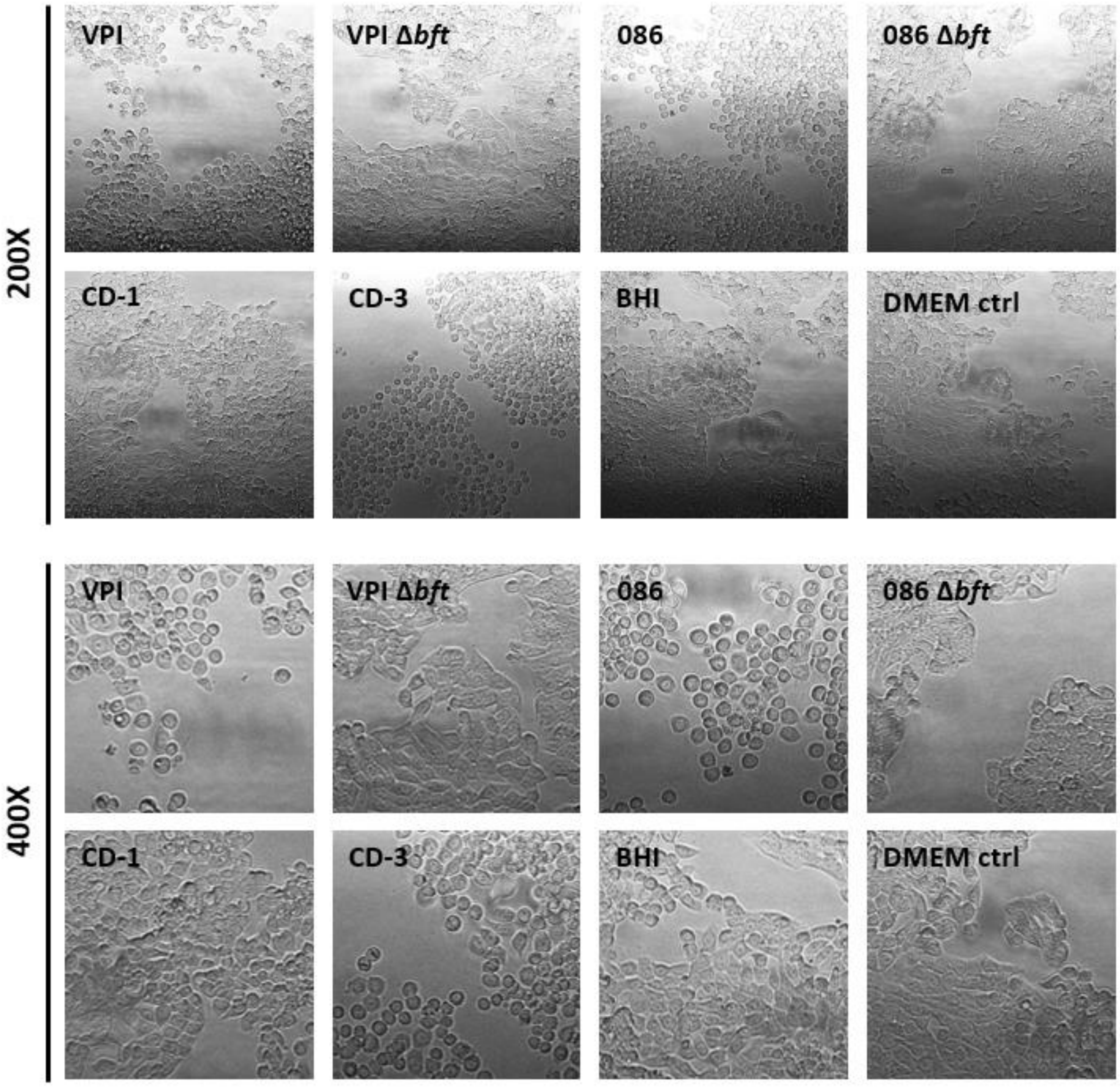

Supplementary Figure 1. HT-29 cell rounding assay for Bft activity. Cell rounding of subconfluent HT-29 cells could be detected in the *B. fragilis* culture supernatants of *bft-1*- and *bft-2*-positive strains, but not in wild-type *bft*-negative strains as well as the respective *bft* deletion mutants.
