# Supplementary material for: Higher Prevalence of Bacteroides fragilis in Crohn’s Disease Exacerbations and Strain-Dependent Increase of Epithelial Resistance
Source: Front Microbiol. 2021 Jun 8;12:598232. doi: 10.3389/fmicb.2021.598232 (PMC8219053; doi:10.3389/fmicb.2021.598232)
Supplement: Supplementary file 2 [file Image_2.pdf]

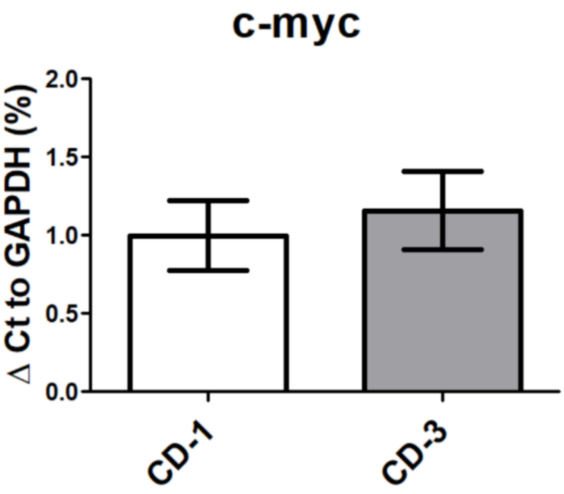

Supplementary Figure 2. Gene expression of *c-myc* in Caco-2 cells differs not significantly when exposed to *B. fragilis* culture supernatants from *bft*-positive CD-3 and *bft*-negative CD-1 strains.
