# Supplementary material for: Higher Prevalence of Bacteroides fragilis in Crohn’s Disease Exacerbations and Strain-Dependent Increase of Epithelial Resistance
Source: Front Microbiol. 2021 Jun 8;12:598232. doi: 10.3389/fmicb.2021.598232 (PMC8219053; doi:10.3389/fmicb.2021.598232)
Supplement: Supplementary file 3 [file Image_3.pdf]

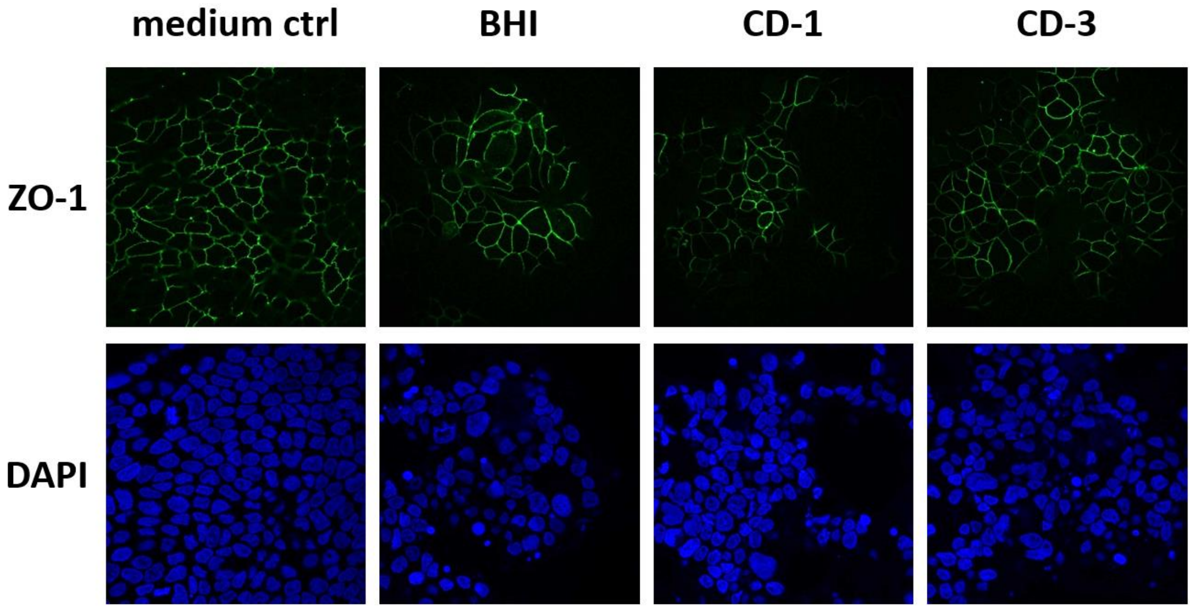

Supplementary Figure 3. Localization of tight junction protein ZO-1. ZO-1 and general cell morphology does not differ between Caco-2 monolayers when exposed to medium only, BHI, and *B. fragilis* culture supernatants from *bft*-positive CD-3 and *bft*-negative CD-1 strains. Magnification: 630x; ZO-1 = Zonulin 1; BHI = brain heart infusion broth
