# Supplementary material for: Higher Prevalence of Bacteroides fragilis in Crohn’s Disease Exacerbations and Strain-Dependent Increase of Epithelial Resistance
Source: Front Microbiol. 2021 Jun 8;12:598232. doi: 10.3389/fmicb.2021.598232 (PMC8219053; doi:10.3389/fmicb.2021.598232)
Supplement: Supplementary file 4 [file Table_1.pdf]

Supplementary table 1

Table S1. Primer sequences for analysis of prevalence and relative abundance.

Gene	Forward primer (5'→3')	Reverse primer (5'→3')	Probe sequence (5'→3')	Label
**gyrB**	GGCGGTCTTCCGGGTAAA	CACACTTCTGCGGGTCTT TGT	TGGCCGACTGCTC	FAM
**bft**	GGGACAAGGATTCTACCA GCTTTATA	ATTCGGCAATCTCATTCA TCATT	CGCAATGGCGAATCCATCA GCTACA	FAM
**ubb**	TGGCTGGACCATAACATTA GAGG	TGCTAAAGTTCGTCCATC TTCCA		
**16S**	CCTACGGGNGGCWGCAG	GACTACHVGGGTATCTA ATCC		
