# Supplementary material for: Higher Prevalence of Bacteroides fragilis in Crohn’s Disease Exacerbations and Strain-Dependent Increase of Epithelial Resistance
Source: Front Microbiol. 2021 Jun 8;12:598232. doi: 10.3389/fmicb.2021.598232 (PMC8219053; doi:10.3389/fmicb.2021.598232)
Supplement: Supplementary file 5 [file Table_2.pdf]

Supplementary table 2

Table S2. Sample origins of isolated *B. fragilis* strains.

**Subject/Origin**	**Year**	**Isolate**	**Disease status**	**Calprotectin**	**Gender**	**Age**
HC-1	2015	*bft-1+ ubb+*	NA	NA	Female	22
HC-2	2015	*bft- ubb-*	NA	NA	Male	NA
ATCC® 25285™	NA	*bft- ubb+*	NA	NA	NA	NA
CD-1	2018	*bft- ubb-*	remission	59 µg/g	Female	41
CD-2	2018	*bft-1+ ubb-*	remission	62 µg/g	Female	29
CD-3	2018	*bft-1+ ubb-*	active	649 µg/g	Male	50
86-5443-2-2 (Pig)	1987	*bft-2+*	diarrheal disease	NA	NA	NA
VPI 13784 (Lamb)	1984	*bft-1+*	diarrheal disease	NA	NA	NA

HC = healthy control, NA = not applicable, *bft* = *B. fragilis toxin*, *ubb* = *ubiquitin*
