# Supplementary material for: Higher Prevalence of Bacteroides fragilis in Crohn’s Disease Exacerbations and Strain-Dependent Increase of Epithelial Resistance
Source: Front Microbiol. 2021 Jun 8;12:598232. doi: 10.3389/fmicb.2021.598232 (PMC8219053; doi:10.3389/fmicb.2021.598232)
Supplement: Supplementary file 6 [file Table_3.pdf]

Supplementary table 3

Table S3. Primer sequences for intercellular junction analysis.

Gene	Sequence ID	Forward primer	Reverse primer
**GAPDH**	NM_002046.4	TGCACCACCAACTGCTTAGC	GGCATGGACTGTGGTCATGAG
**18S**	M10098	GTAACCCGTTGAACCCCATT	CCATCCAATCGGTAGTAGCG
**ZO-1**	NM_003257.3	AGGGGCAGTGGTGGTTTTCTGTTCTTTC	GCAGAGGTCAAAGTTCAAGGCTCAAGAGG
**Occludin**	NM_002538.2	TCAGGGAATATCCACCTATCACTTCAG	CATCAGCAGCAGCATGTACTCTTCAC
**E-cadherin**	NM_004360.3	CACCTGGAGAGAGGCCGCGT	AACGGAGGCCTGATGGGGCG
**β-catenin**	NM_001904.3	GTGCTATCTGTCTGCTCTAGTA	CTTCCTGTTTAGTTGCAGCATC
**Claudin-1**	NM_021101.4	GGGCTGCAGCTGTTGGGCTT	GGGTTGCTTGCAATGTGCTGCT
**Claudin-2**	NM_020384.3	AACTACTACGATGCCTACC	GAACTCACTCTTGACTTTGG
**Claudin-3**	NM_001306.3	TTCATCGGCAGCAACATCATC	CGCCTGAAGGTCCTGTGG
**Claudin-4**	NM_001305.4	ACAGACAAGCCTTACTCC	GGAAGAACAAAGCAGAG
**Claudin-7**	NM_001185022.1	GCCATACCAGGAGCAAGC	GGAGACGACAAAGTGAAGA
**Claudin-12**	AJ250713.1	CTCCCCATCTATCTGGGTCA	GGTGGATGGGAGTACAATGG
**c-myc**	NM_002467.5	AAA GGC CCC CAA GGT AGT TA	GCA CAA GAG TTC CGT AGC TG
